# Endothelial dysfunction in vascular complications of diabetes: a comprehensive review of mechanisms and implications

**DOI:** 10.3389/fendo.2024.1359255

**Published:** 2024-04-05

**Authors:** Dong-Rong Yang, Meng-Yan Wang, Cheng-Lin Zhang, Yu Wang

**Affiliations:** ^1^Department of Endocrinology and Metabolism, Shenzhen University General Hospital, Shenzhen, Guangdong, China; ^2^Department of Pathophysiology, Shenzhen University Medical School, Shenzhen, Guangdong, China

**Keywords:** diabetes, endothelial dysfunction, vascular diseases, atherosclerotic, therapies

## Abstract

Diabetic vascular complications are prevalent and severe among diabetic patients, profoundly affecting both their quality of life and long-term prospects. These complications can be classified into macrovascular and microvascular complications. Under the impact of risk factors such as elevated blood glucose, blood pressure, and cholesterol lipids, the vascular endothelium undergoes endothelial dysfunction, characterized by increased inflammation and oxidative stress, decreased NO biosynthesis, endothelial-mesenchymal transition, senescence, and even cell death. These processes will ultimately lead to macrovascular and microvascular diseases, with macrovascular diseases mainly characterized by atherosclerosis (AS) and microvascular diseases mainly characterized by thickening of the basement membrane. It further indicates a primary contributor to the elevated morbidity and mortality observed in individuals with diabetes. In this review, we will delve into the intricate mechanisms that drive endothelial dysfunction during diabetes progression and its associated vascular complications. Furthermore, we will outline various pharmacotherapies targeting diabetic endothelial dysfunction in the hope of accelerating effective therapeutic drug discovery for early control of diabetes and its vascular complications.

## 1 Introduction

**Figure 1:**
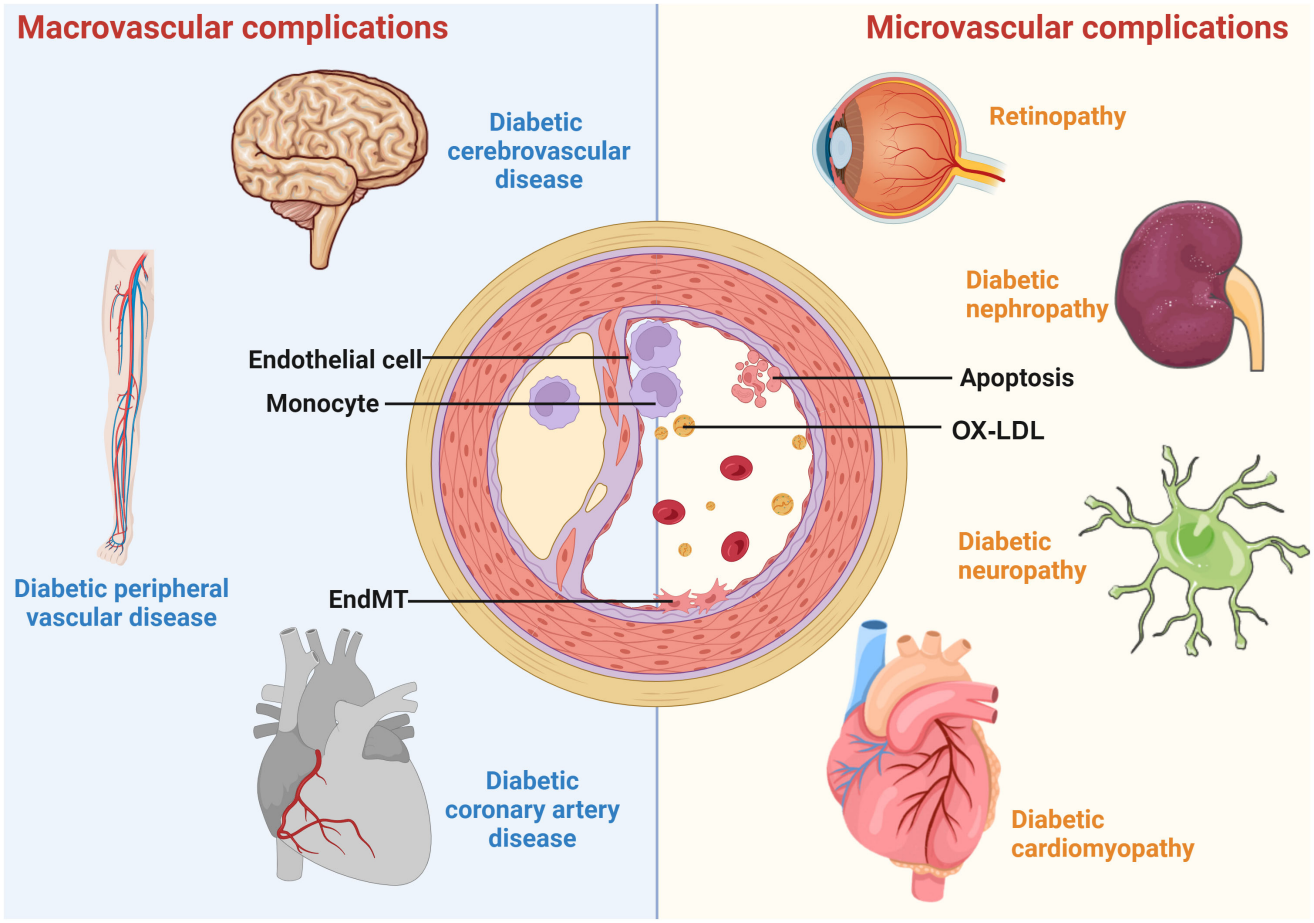
A schematic overview of diabetic vascular complications. Diabetic vascular complications include macrovascular complications (diabetic coronary artery disease, cerebrovascular disease, and peripheral vascular disease) and microvascular complications (diabetic retinopathy, nephropathy, cardiomyopathy, and neuropathy). Continued exposure to risk factors causes endothelial dysfunction, leading to lipid retention in the endothelium. Monocytes differentiate into macrophages, internalize modified lipoproteins, and form foam cells. Activated foam cells induce inflammation by secreting cytokines through several downstream signals. In addition, endothelial cells undergo EndMT, cell death, etc., further exacerbating the atherosclerotic process.

Diabetes is a chronic and severe metabolic disorder characterized by consistently high blood sugar levels. According to the International Diabetes Alliance statistics, the number of individuals living with diabetes worldwide in 2021 is around 537 million, with an estimated increase to 783 million in 2045 (1). The health burden of diabetes is largely due to diabetic vascular complications. Diabetic vascular complications include macrovascular complications, such as coronary artery disease (CAD), cerebrovascular disease, peripheral artery disease (PAD), and microvascular diseases, including diabetic nephropathy (DN), diabetic retinopathy (DR), diabetic neuropathy (DPN), and cardiomyopathy (**Figure 1**). These complications are critical factors that contribute to a huge burden on individuals with diabetes (2).

**Figure 2:**
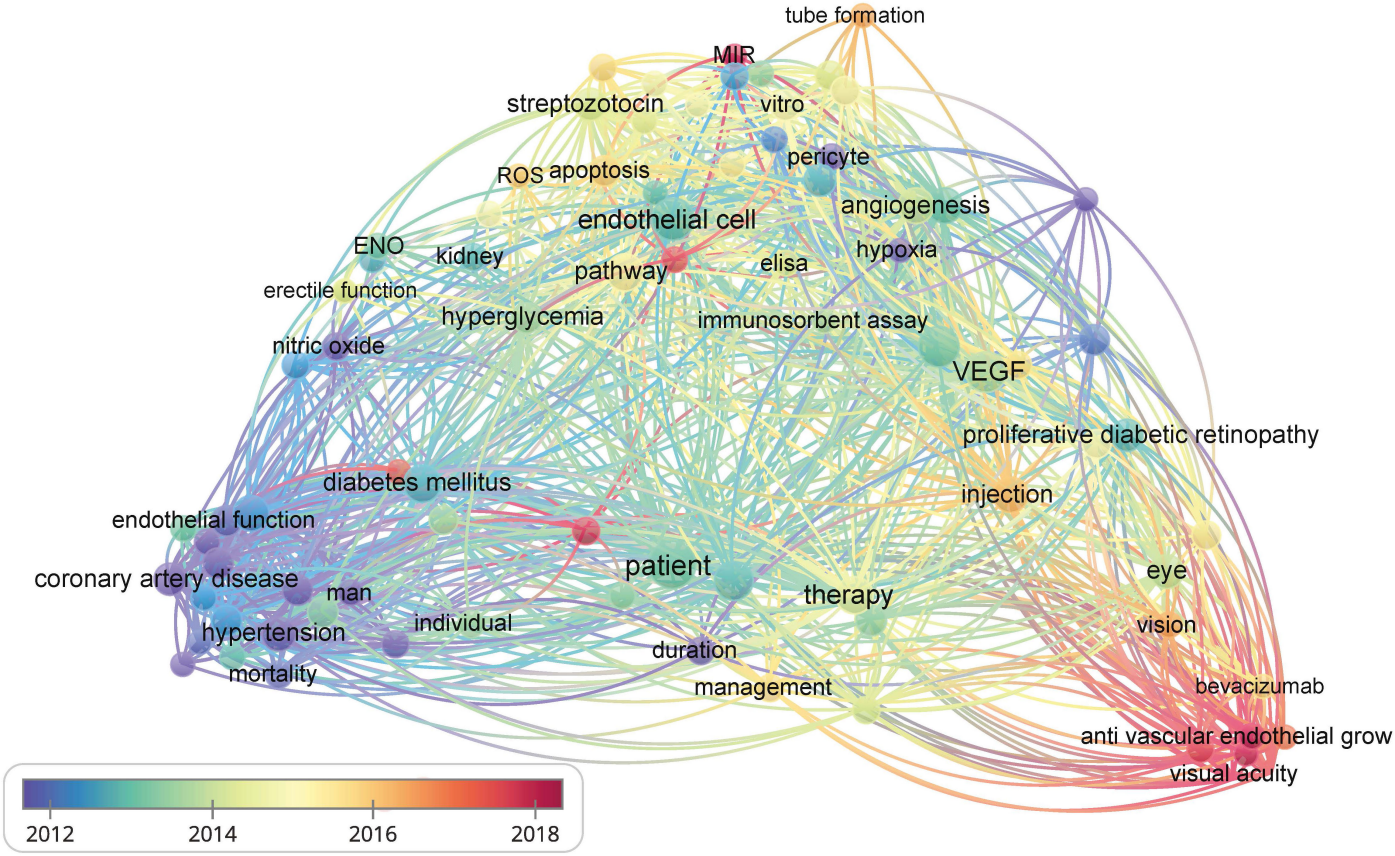
The bibliometric data obtained using VOSviewer. It shows the frequency of keywords in the literature related to diabetes and its vascular complications. The color of dots and lines represent the average year of publication for the corresponding term.

The vascular endothelium is a continuous lining within the vascular system that is responsible for maintaining vascular tension, angiogenesis, and hemostasis. Additionally, it provides crucial antioxidant, anti-inflammatory, and antithrombotic surfaces. Endothelial dysfunction can be narrowly defined as a decrease in vasodilation capacity or, more broadly, as any changes affecting endothelial homeostasis function. Although the interaction between endothelial dysfunction and vascular complications of diabetes is not fully understood, the existing view shows that a high glucose environment leads to the impairment of endothelium-dependent vasodilation capacity by increasing oxidative stress and inflammation, reducing vasodilation factor activity, increasing reactive oxygen species (ROS) production, promoting the uncoupling of endothelial nitric oxide synthase (eNOS), and so on (3). Endothelial dysfunction is also an important early indicator of atherosclerosis (AS) and identifies patients at a higher vascular risk, providing a “barometer” for vascular health (4). Therefore, endothelial dysfunction can be an early event in the vascular complications of diabetes. Understanding the importance of endothelial dysfunction is particularly crucial for controlling diabetic vascular complications and lowering the prevalence of diabetic vascular diseases. Additionally, researchers have established the detrimental role of endothelial dysfunction in the onset and development of serious diabetic microvascular diseases, such as heart failure, cognitive decline, and worsening metabolic dysfunction, in addition to vision loss, renal insufficiency, and neuropathy (5). We further confirmed the research keywords related to diabetic vascular complications through visual analysis and identified endothelial function as a hot topic in this field. Therefore, impaired endothelial function could become a promising target for preventing vascular complications in patients with diabetes, especially in DR and CAD (**Figure 2**). However, there has not been an updated review on the mechanisms and therapies of diabetic vascular complications, especially focusing on endothelial dysfunction. We aim to provide a comprehensive summary of diabetic endothelial dysfunction, which may offer a deeper insight into endothelial dysfunction to not only help prevent the deterioration of diabetes and its vascular complications but also accelerate the process of developing more beneficial therapies.

In this review, we present the research progress on the mechanisms of endothelial dysfunction in diabetes and its vascular complications, mainly focusing on inflammation, oxidative stress, endothelial-to-mesenchymal transition (EndMT), and cell death. We also summarized the effects and limitations of common hypoglycemic agents and novel regulators on the vascular endothelium.

## 2 Mechanisms of diabetic endothelial dysfunction

### 2.1 Inflammation

Chronic, low-grade inflammation is widely considered to be a significant factor in the development of diabetes. Hyperglycemia can activate various pathways and pro-inflammatory factors that induce endothelial dysfunction. It triggers the nonclassical nuclear factor kappa-B (NF-κB) signaling pathway, produces cytokines and chemokines, promotes inflammation, and damages β cellular function (6). Many early findings have identified the proinflammatory effect of NF-κB, tumor necrosis factor-α (TNF-α), and interleukin-6 (IL-6) on endothelial dysfunction in diabetes (3). TNF-α in particular can activate critical intracellular signaling molecules in various inflammatory signaling systems, such as c-Jun amino-terminal kinases (JNK) and IkappaB kinase beta (IKKβ) (7, 8), leading to impaired insulin action. Under normal conditions, insulin activates the phosphatidylinositol 3-kinase (P13K) pathway, which phosphorylates eNOS at Ser^1177^ in endothelial cells (ECs) to produce nitric oxide (NO) to maintain normal endothelial function (9). In an insulin-resistant state, this pathway is disrupted, leading to the imbalance between the phosphoinositide-3 kinase (PI-3K)/protein kinase B (Akt/PKB) and mitogen-activated-protein kinase (MAPK)/extracellular signal-regulated kinases (ERK) pathways. This will eventually disrupt vascular homeostasis. Activation of the MAPK pathway can release inflammatory mediators such as vascular cell adhesion protein 1 (VCAM-1), intercellular adhesion molecule 1 (ICAM-1), E-selectin, and plasminogen activator inhibitor-1 (PAI-1), which can compromise normal endothelial function (10). In addition, in experimental models of cardiovascular end-organ damage associated with diabetes, ANG- (1–7) has been shown to act as an anti-inflammatory agent (11). It regulates the expression of VCAM-1 induced by ANG II by reducing the nuclear translocation of NF-κB in ECs (12). WNT5A, a ligand of the Wnt signal pathway, gained attention for its pro-inflammatory effects and its role in the pathogenesis of diabetic complications (13). Researchers discovered that SETD8 (SET8) can modulate WNT5A function and control the production of proinflammatory enzymes and endothelial adhesion. Overexpression of SETD8 reduced inflammation by decreasing the activation of the NOD-like receptor pyrin domain 3 (NLRP3) inflammasome and the expression of microtubule-affinity regulating kinase 4 caused by hyperglycemia in human umbilical vein endothelial cells (HUVECs) (14, 15). Further to this, vascular endothelial growth factor (VEGF) levels change significantly in diabetic patients and play a vital role in diabetic microvascular complications (16). Activation of vascular endothelial growth factor receptor 1 (VEGFR1) in ECs attracts inflammatory cells to white adipose tissue in type 2 diabetes mellitus (T2DM). This effect can be alleviated by VEGF-B deficiency targeting the VEGFR1-EC axis (17).

Intracellular hyperglycemia can increase production of advanced glycation end products (AGEs), promoting the interaction of AGEs and the advanced glycosylation end product-specific receptor (AGER, also known as RAGE). This receptor acts as a pattern-recognition receptor that initiates specific cell signaling (18). AGE-RAGE is associated with multiple signal transduction pathways that promote atherosclerotic formation. This includes increased expression of VCAM-1, macrophage inflammatory protein-1 (MIP-1), matrix-metalloproteinase 9 (MMP9), interleukin-1β (IL-1β), and TNF-α, which mediate leukocyte adhesion and vascular inflammatory responses (19). Serum albumin glycosylation in a high glucose environment forms AGE-modified human serum albumin (AGE-HSA). In diabetic patients, it can activate the production of macrophages C-C chemokine ligand 5 (CCL-5) and IL-8, driving an increase in the inflammatory response. Inflammatory cytokines may increase the total amount of AGE-HAS in the body, leading to a vicious cycle (20).

In addition to the pathways mentioned above, hyperglycemia also genetically regulates inflammation in endothelial dysfunction. By partially suppressing signal transduction and activators of transcription 1 (STAT1), hyperglycemia upregulates the expression of proinflammatory target genes, including *CCL5*, *CXCL10*, and *ICAM1.* This causes tumor necrosis factor-like weak inducer of apoptosis (TWEAK) to demonstrate its pro-atherosclerotic tendency (21). Moreover, Zhu et al. found that, when exposed to high glucose levels, the activation of the mechanosensory ion channel Piezo1 upregulates genes involved in inflammatory pathways (such as *IL1B*). This, in turn, increases the risk of pathological mechanics-induced thrombosis by enhancing the prothrombin reaction of platelets, erythrocytes, and neutrophils, ultimately leading to disrupted blood flow (22). In this disordered blood flow state, ECs are affected by turbulent mechanical stimulation, which causes an increase in Piezo1 expression beyond the threshold, which activates the NF-κB pathway and finally leads to atherosclerosis (23).

### 2.2 Oxidative stress

Oxidative stress occurs when the balance between intracellular pro-oxidation and antioxidant systems is disrupted. In diabetic ECs, intracellular hyperglycemia can increase mitochondrial ROS (mtROS) generation, which accelerates endothelial dysfunction. ROS were thought to be primarily produced by the NADPH oxidase (NOX) family in phagocytic cells (24, 25). NOX1 and NOX4 were upregulated in aortas and mesenteric arteries of T2DM mice (26). Enhanced NOX4 activity and expression were also found in rats with type 1 diabetes (27). Both may lead to an increase in ROS. Angiotensin converting enzyme-2 (ACE2) promotes angiotensin (ANG)-(1-7)-dependent vasodilation and may inhibit NOX-induced ROS production in ECs. Therefore, in diabetes-induced kidney injury, mice deficient in ACE2 exhibit an increase in oxidative stress (12, 28). Ang-(1-7) treatment also attenuated the elevation of renal NOX activity in the kidneys of diabetic spontaneously hypertensive rats, reducing the degree of hyperglycemia. It significantly prevented the diabetes-induced reduction in catalase activity, as well as the reduction in PPAR-gamma mRNA and protein levels *in vitro* (27, 29). High levels of ROS generation or low antioxidant activity can result in endothelial mitochondria dysfunction (30). Mitochondrial calcium uptake 1 (MICU1) in the mitochondrial inner membrane regulates calcium uptake and maintains calcium homeostasis. Endothelial MICU1 expression decreased in diabetes and other vascular illnesses including diabetic cardiomyopathy. Inhibiting MICU1 resulted in an increase in mitochondrial ROS production (31). The impaired mitochondria isolated from hyperglycemia-treated cells showed increased palmitoylcarnitine oxidation, decreased pyruvate oxidation, and Uncoupling protein 2 (UCP2). These findings suggest that hyperglycemia may cause excessive mitochondrial ROS formation by increasing fatty acid oxidative damage or uncoupling the mitochondrial oxidative phosphorylation system (32). ECs motility, proliferation, angiogenesis, and death are all reliant on mitochondrial metabolism (33). Therefore, the damaged mitochondrial metabolism induced by hyperglycemia may impact normal EC function, leading to endothelial dysfunction.

Sun indicated that oxidative stress activates Sirtuin 1 (SIRT1), which deacetylates the forkhead box transcription factor O1 (FOXO1) and traps it in the nucleus, enhancing its transcriptional activity. FOXO1 enhancement may inhibit the repair of glycolysis-dependent oxidative DNA damage, which can worsen endothelial oxidative damage (34). However, another study showed that SIRT1 promotes insulin secretion and pancreatic beta cell survival by interacting with FOXO, which can alleviate diabetes (35). Additionally, restoring aortic SIRT1 levels can significantly improve endothelial dysfunction and vascular compliance by enhancing eNOS activity and inhibiting NOX-associated oxidative stress (36). SIRT1 can improve vascular function in diabetes by deacetylating the 66-kDa Src homology 2 domain-containing protein (p66Shc) and reducing ROS production (37). The differences in experimental materials, the degree of FOXO activation, and the complicated functions of SIRT1 may lead to the differences in the above research findings. Besides, healthy ECs normally undergo a metabolic shift to glycolysis to facilitate prompt DNA repair responses after H2O2 treatment. However, overactivation of FOXO1 in hyperglycemia can cause DNA damage, leading to endothelial dysfunction (34). Consequently, the function of SIRT1 and FOXO in diabetic endothelial function needs further exploration.

### 2.3 Endothelial-to-mesenchymal transition

EndMT is a process in which ECs transform into mesenchymal stem cells under certain pathological situations, such as high oxidative stress, disturbed metabolism, hypoxia, and shear stress force (38). Notably, EndMT has been discovered to exist in a majority of diabetic complications (39). If a significant proportion of ECs undergo EndMT, this may lead to an endothelial layer disruption resulting in plaque erosion and finally accelerating the progression of atherosclerotic disease (40).

Hyperglycemia activates many downstream signaling pathways to initiate EndMT in diabetic Ecs (39). These signaling pathways can be divided into TGF-β signaling and non-TGF-β signaling (41). TGF-β signaling contains canonical Smad2/3-mediated pathways and noncanonical Smad2/3-independent signaling pathways such as MAPK, RhoA, and c-Jun NH2-terminal kinases (JNK). Non-TGF-β signaling mainly includes Notch signaling, the canonical Wnt pathway, and other regulatory mechanisms mediated by cytokines and some inflammatory mediators (42). Of all the signaling pathways, EndMT is thought to be most significantly influenced by the TGF-β signaling pathway, specifically the canonical downstream Smad pathway (43).

Hyperglycemia induces EndMT by directly increasing TGF-β signaling and non-TGF-β signaling. TGF-β signaling is activated by hyperglycemia as a part of EC metabolic memories, which can cause EndMT in Ecs even after the culture condition is changed to normal glucose levels (44). By directly increasing the production of angiotensin II (Ang II), hyperglycemia causes EndMT in primary human aortic endothelial cells, which damages the endothelium (45). The negative effects of Ang II/AT1 pathway in Ecs can be counteracted by the Ang (1-7)/Mas pathway. The equilibrium of Ang II and Ang (1-7) might be a crucial factor in the pathophysiological events that result in endothelial dysfunction (12, 46). Additionally, the crosstalk of the TGF-β pathway and Wnt/β-catenin signaling also regulates EndMT and contributes to endothelial problems in diabetic situations, since increased β-catenin expression enhances TGF-β sensitivity in hyperglycemic situations (47).

Except for the direct role of hyperglycemia in EndMT, the higher context of inflammation and oxidative stress can regulate EndMT in many ways. NF-κB activation-mediated EndMT can be induced by IL-1β plus TGF-β2 or TNF-α and IL-6 stimulation of ECs (48, 49). EndMT can also be induced by oxidative stress. The expression of endothelial and fibrotic markers changed with increasing dosages of H_2_O_2_ (0.1–10 μM) and initiated the transformation process in HUVECs. This was accompanied by increased expression of TGF-β1 and TGF-β2 and was found to be reliant on activin receptor-like kinase 5 (ALK5) expression, Smad3 activation, and NF-κB activity (50). Moreover, SIRT1 can block EndMT in human ECs via deacetylating the Smad4 pathway (51). AMP-activated protein kinase (AMPK) activates SIRT1, which can inhibit Smad2/3 nuclear translocation, thereby decreasing TGFβ-induced EndMT. This suggests that AMPK may be involved in EndMT-induced diabetic endothelial damage (52, 53).

### 2.4 Cell death

Cell death can be classified into two categories: programmed and accidental. The main form of accidental cell death is called necrosis, where intracellular contents are released because of the rupture of the plasma membrane (54). Regulated cell death caused by an intracellular program is referred to as programmed cell death and includes apoptosis, autophagy, pyroptosis, ferroptosis, and necroptosis. Regulated cell death influences various pathophysiological situations, including inflammation, immunology, tissue homeostasis, and cell growth. Diabetic endothelial injury is significantly associated with cell death (55).

Apoptosis is a crucial feature of vascular injury under some circumstances, such as high levels of ox-LDL, AGE, oxidative stress in the blood, and disturbed blood flow (3). A recent study reported that TNF-related apoptosis-inducing ligand-deficient diabetes mice fed a high-fat diet displayed accelerated AS. These mice also showed more widespread necrotic cores in plaques and enhanced macrophage infiltration and apoptosis in islets (56). Endothelial WW domain-containing E3 ubiquitin protein ligase 2 (WWP2) can preserve cell survival and prevent apoptosis by targeting DEAD-box helicase 3 X-linked (DDX3X) for K63-linked polyubiquitination and proteasomal degradation, thus preventing endothelial injury (57). In addition, knocking down nuclear factor erythroid 2-related factor 2 (Nrf2) increases apoptosis and hinders tube formation in endothelial progenitor cells (EPCs) sourced from healthy donors and wild-type mice. However, overexpression of Nrf2 reduced apoptosis and restored tube formation in EPCs obtained from diabetic patients and *db/db* mice (58).

Autophagy is a life-sustaining process that helps maintain endothelial function (59). It can be classified into two categories: selective and non-selective. Selective autophagy such as mitophagy, lysophagy, and ER-phagy specifically targets and engulfs substrates. High glucose levels suppress the CAV1-CAVIN1-LC3B-mediated autophagic degradation of CAV1, a component of caveolae. This process promotes the transcytosis of LDL across ECs, ultimately resulting in the subendothelial retention of atherogenic lipids (60). The impairment of autophagosome-lysosome fusion and the inhibition of autophagy-related 14 (Atg14) expression, meditated by FOXO1, leads to autophagic apoptosis in ECs induced by AGEs (59). Most studies have shown that mitophagy protects hyperglycemia-treated ECs. To counteract mitochondrial ROS and prevent aging, high glucose increases mitophagy in human aortic ECs (61). Hyperglycemia dramatically inhibited mitophagy in HUVECs by downregulating PTEN-induced kinase 1 (PINK1), Parkin, LC3 II, Beclin-1, and *autophagy-related gene 5* (*ATG5)* (62). Another study found that diabetic mice have higher levels of PINK1 and Parkin in the vascular wall. The PINK1-Parkin pathway induces mitophagy to prevent metabolic stress-induced endothelium damage (63). However, increases or decreases in PINK1 and Parkin conflict. The difference may be attributed to empirical materials and the high-glucose duration of treatment. Hence, more research is required to confirm the precise changes in Parkin and PINK1 levels.

Ferroptosis is a type of regulated necrosis that depends on iron and results from significant damage to cell membranes due to lipid peroxidation. It typically occurs alongside significant iron accumulation and lipid peroxidation during cell death (64). Vascular ECs exposed to high glucose levels *in vitro* exhibited higher levels of ferroptosis-associated proteins (65). The expression of heme oxygenase 1 (HMOX1) markedly increased in diabetic mice and diabetic ECs treated with high glucose and high lipid. HMOX1 upregulation is associated with increased ferroptosis in diabetes-induced endothelial injury. Suppression of HMOX1 can effectively rescue the negative effects of high glucose and high lipids by rebalancing iron and oxidative stress and blocking excessive ferroptosis (66). Moreover, glutathione peroxidase 4 (GPX4), an antioxidant enzyme, facilitates the removal of excess lipid peroxides. It’s a crucial upstream regulator of ferroptosis (67). The intracellular GPX4 reduction in hyperglycemia environments then leads to an overabundance of ROS and Fe^2+^ in various cells, including ECs. These elements are considered critical factors in the initiation of ferroptosis (68). Hyperglycemia and IL-1β leads to ECs ferroptosis and dysfunction by downregulating xCT (the substrate-specific subunit of system Xc^-^) expression through activation of p53-XcT-glutathione (GSH) axis (69).

Pyroptosis is a recently discovered form of programmed cell death that involves an early disruption of the plasma membrane, leading to the extracellular leakage of intracellular contents (70). In recent years, researchers have extensively studied the connection between pyroptosis and diabetic microvascular complications. In diabetic retinopathy, treatment of human retinal microvascular endothelial cells (HRMECs) with hyperglycemia resulted in decreased cell viability and increased caspase1 activity, indicating hyperglycemia’s induction of HRMECs pyroptosis (71). In addition, the pyroptotic pathway mediated by caspase1-GSDMD was activated by hyperglycemia in glomerular endothelial cells (GECs), which induced the development of diabetic nephropathy (72).

**Figure 3:**
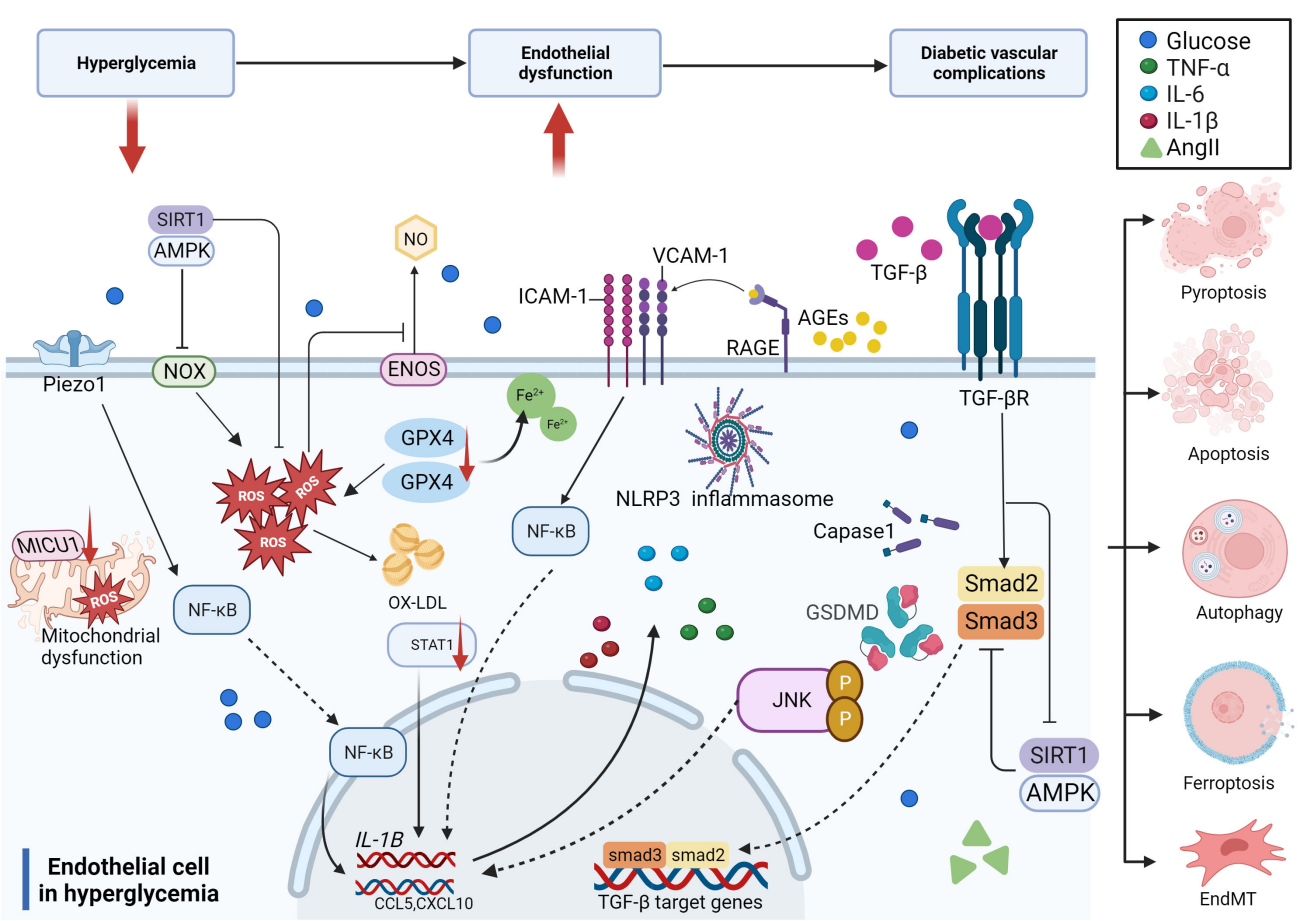
Molecular mechanisms of diabetic endothelial dysfunction. The mechanisms of diabetic endothelial dysfunction are complicated and include oxidative stress, inflammation, EndMT, and cell death. Most of these related factors work together to accelerate the process of diabetic endothelial dysfunction through different signaling pathways, which ultimately lead to diabetic vascular complications.

In summary, hyperglycemia and related risk factors, including high lipids and a disordered blood flow state, contribute to endothelial dysfunction through several pathways, such as increased inflammation, reactive oxygen species (ROS) production, EndMT, and cell death. The compromised endothelium is responsible for initiating vascular dysfunction, which may progress into more severe vascular problems without continuous glucose control and treatment over time (**Figure 3**).

## 3 Diabetic macrovascular complications

Diabetic macroangiopathy mainly contains AS in large and medium arteries (aorta, coronary, basilar, and peripheral arteries), leading to vascular disorders including coronary artery disease (CAD), peripheral artery disease (PAD), and cerebrovascular disease. AS is characterized by the accumulation of fatty and/or fibrous material in the intima. Endothelial dysfunction is required for the initiation of AS, which has been identified as a major cause of diabetic macrovascular complications (73).

### 3.1 Diabetic atherosclerosis

Previous research has demonstrated that diabetes mellitus and AS share the same endothelial pathological response (74). As mentioned above, hyperglycemia can lead to endothelial dysfunction via various mechanisms. Endothelial dysfunction is indeed the key factor in triggering AS, so diabetes can promote the onset and development of AS.

**Figure 4:**
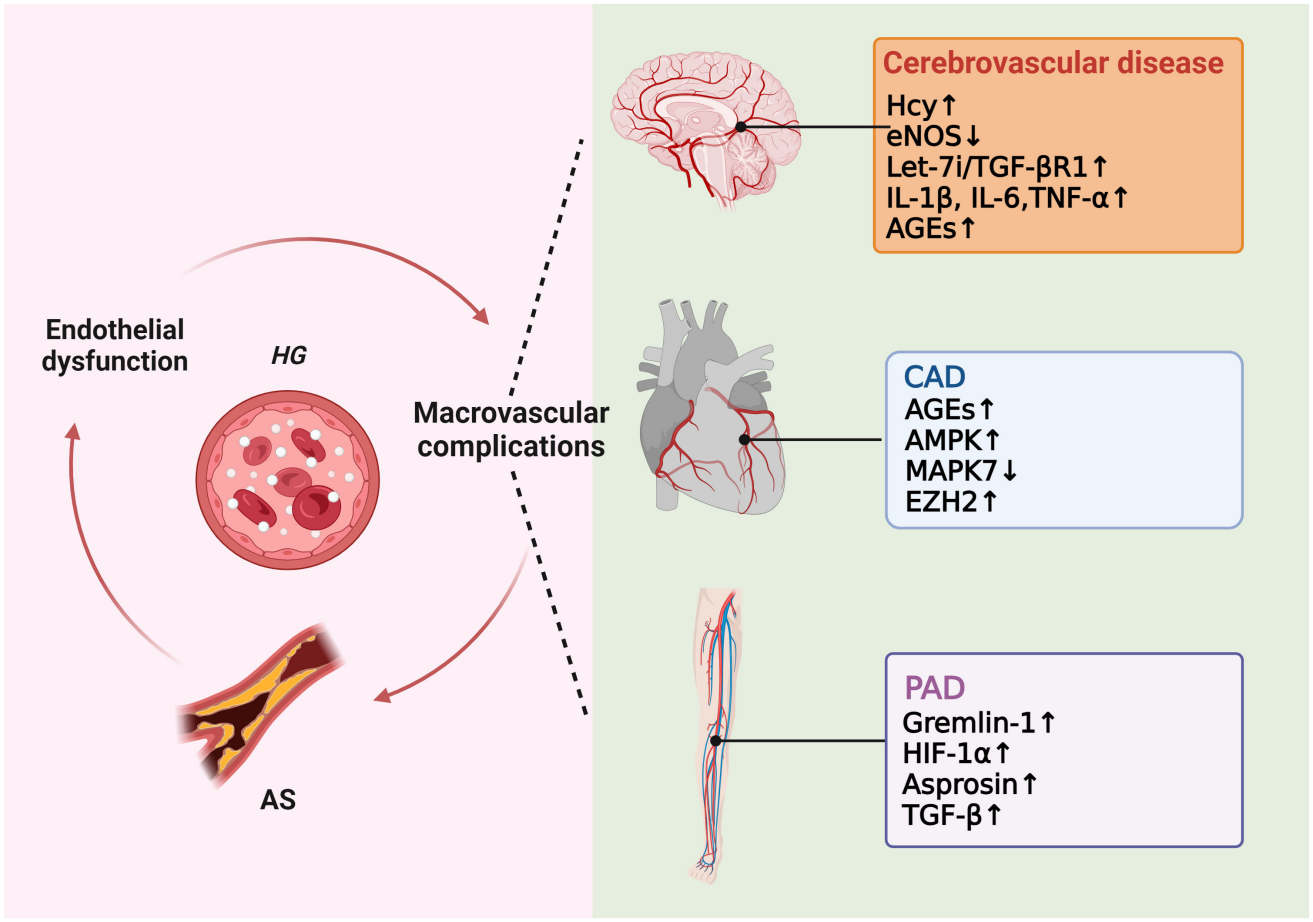
Molecular mechanisms of diabetic macrovascular complications. Hyperglycemia leads to endothelial dysfunction, atherosclerosis (AS), and diabetic macrovascular disorders, including coronary artery disease (CAD), cerebrovascular disease, and peripheral vascular disease. These conditions affect each other in a vicious cycle if they are not treated appropriately.

As previously mentioned, many studies have proved that high glucose induces ROS production, which in turn causes AS. Excessive ROS can accelerate AS by directly activating proinflammatory pathways and indirectly by increasing the formation of ox-LDL, promoting insulin resistance, activating ubiquitination-related pathways, and decreasing the activation of adiponectin, AMPK, and eNOS (24). In hyperglycemic macrovasculature damage, ROS and inflammatory cytokines cause signaling dysregulation and EndMT. EndMT produces mesenchymal cells that express and deposit ECM proteins, accelerating the development of AS by acting as a support for developing plaque (75, 76). Hyperglycemia-induced TGF-β signaling activation also prompts EndMT and AS (77). Eventually, atherosclerotic plaque encroaches on the artery lumen, causing flow-limiting lesions and serious vascular consequences. Thus, without diabetes control and therapy, a damaged endothelium will worsen, causing AS and other vascular complications in diabetes (**Figure 4**).

### 3.2 Diabetic coronary artery disease

Despite the significance of all T2DM complications, cardiovascular disease (CAD) remains the leading cause of morbidity and mortality in individuals with diabetes (78). It is worth highlighting that people with T2DM have a higher risk of developing cardiovascular diseases compared to those without diabetes, with the risk being two to four times higher (79).

CAD and its related complications are believed to be strongly associated with oxidative stress (80). As stated above, diabetics have high oxidative stress due to the inequality between the production of ROS and the protection systems of both enzymatic and non-enzymatic antioxidants. This can lead to endothelial dysfunction and AS of the coronary arteries (81). CAD has been linked to AGEs accumulation in patients diagnosed with T2DM (82). Diabetic patients with obstructive CAD have higher serum AGEs concentrations compared to T2DM patients without obstructive CAD (83). In comparison to those with CAD alone, patients with CAD and T2DM had impaired endothelium-dependent vasodilation and enhanced mitochondrial H_2_O_2_ production and AMPK activation in primary human saphenous vein endothelial cells mediated by mtROS (84). Additionally, the elevated plasma concentration of TNF-α and its receptor observed in the coronary arterioles of *db/db* mice enlightens the role of inflammation in DM patients with CAD (85). With the increase in intima/media thickness ratio in CAD patients, atheroprotective Mitogen-activated protein kinase 7 (MAPK7) expression decreased, whereas Zeste Homolog 2 (EZH2) expression increased. MAPK7-EZH2 reciprocity may indicate an autoregulatory feedback loop in ECs that maintains endothelial homeostasis. Disruptions in this relationship that promotes EZH2 expression can cause endothelial dysfunction and EndMT, aggravating the severity of human CAD (86).

### 3.3 Diabetic cerebrovascular disease and stroke

Numerous studies have shown increased infarct size, edema, and hemorrhage in hyperglycemic animals as compared to their normal counterparts (87). Ischemic stroke is the most common cerebrovascular complications of diabetes (88). Stroke related to diabetes is often caused by extracranial carotid artery disease and intracranial large and small vessel diseases. It is the second highest cause of death among patients with T2DM (79).

Endothelial dysfunction aggravated by diabetes is an essential factor in stroke development in diabetic patients and may contribute to endothelial dysfunction after cerebral ischemia (88, 89). Additionally, in the normal cerebral microcirculation and major cerebral arteries, endothelium generates NO both basally and in response to various vasoactive stimuli. However, in diabetic encephalopathy, there is a decrease in the expression of VEGF and eNOS. This leads to a diminished vascular autoregulatory response and impaired cerebral artery endothelial function, further worsening diabetic cerebrovascular disease (90). As a risk factor for cerebrovascular diseases in DM patients, serum homocysteine (Hcy) increases significantly in T2DM patients (91). Hcy harms vascular endothelium directly by oxidative stress and endoplasmic reticulum stress or indirectly by the cytokine and the immune response (92, 93). Ischemic stroke initiates a let-7i/TGF-βR1 double-negative feedback loop that causes EndMT in ECs and vascular fibrosis. This suggests that EndMT could be a target for treating cerebral vascular fibrosis (94).

Diabetic macroangiopathy and microangiopathy exacerbate each other. Macrovessel obstruction may lead to brain perfusion deficiency and microvascular diseases. Microvascular dysfunction also affects macrovessel collateral circulation, increases the risk of stroke, and worsens prognosis (95). As the main part of the cerebral microvascular system, blood-brain barrier (BBB) dysfunction may worsen cerebral macroangiopathy dysfunction by affecting cerebral microangiopathy indirectly. Higher expression of proinflammatory factors such as IL-1β, IL-6, and TNF-α and accumulation of AGEs in a diabetes-induced inflammatory environment can all harm the BBB (95, 96). Tight junctions (TJs) intercellularly among ECs effectively close gaps between adjacent cells. They are reduced by DM in the parenchymal blood vessels, which disrupts the BBB and makes it easier for albumin and inflammatory factors to enter the brain (97).

### 3.4 Diabetic peripheral vascular disease

Peripheral arterial disease (PAD), an occlusive atherosclerotic disease in the arteries of the lower limbs, is the most common initial manifestation of cardiovascular disease in T2DM (98). Despite this, it is frequently overlooked. Patients with diabetes may experience diabetic foot ulcers (DFUs) and PAD as two typical displays of diabetic peripheral vascular disease. DFU is a common cause of lower limb loss by amputation and are long-term consequences of diabetes. Diabetes-related mortality has DFUs as a major contributing factor (99).

Bapir et al. assessed endothelial function by flow-mediated dilation (FMD). They discovered that the femoral artery (FA)-FMD was considerably lowered in T2DM patients compared to healthy individuals, indicating a limb arterial endothelial impairment of T2DM patients (100). The levels of AGEs, MDA, and TNF-α in diabetic patients with DFU is significantly higher. These elements are necessary for the secretion of soluble vascular endothelial growth factor receptor-1 (sVEGFR-1), which ultimately decreases the expression of VEGF compared to the diabetic group without DFU and interrupts the wound healing process in diabetic patients (101). Other scientists found that VEGF, Gremlin-1, and HIF-1α increased in DFU patients. As an oxygen concentration-dependent transcription factor, HIF-1α can control a variety of target genes, most notably VEGF. Gremlin-1 is a coactivator of VEGF and can induce microangiogenesis, which is a process involved in the pathogenesis of diabetic lower limb ulcers (102). Consequently, these all lead to more severe DFU. Metabolomics studies have shown that amino acid metabolism disorders are the main metabolic hallmarks of PAD patients. In T2DM patients with lower extremity PAD, asprosin levels are very high, which promotes EndMT by activating the TGF-β pathway (103). Neutrophils are more suitable for neutrophil extracellular trap (NET)osis in diabetic wounds. NETosis delays diabetic wound healing by inducing EndMT via the Hippo-Yes-associated protein (YAP) pathway. The Hippo pathway mediates ECs dysfunction and regulates angiogenesis by inducing EndMT via the transcription factor Smad2 (104).

## 4 Diabetic microvascular complications

### 4.1 Diabetic nephropathy

Hyperglycemia is a key factor in the onset of DN and has been identified as one of the significant long-term consequences of DM that affects the microvasculature (105, 106).

Glomerular endothelial cells (GECs) are specialized vascular cells found in the kidney that help maintain renal homeostasis by forming the walls of the glomerular tufts. The inflammatory environment of diabetes can result in GECs dysfunction, leading to proteinuria and renal fibrosis. The GECs of diabetic mice have a high representation of genes involved in the oxidative stress pathway. Hyperglycemia raises leucine-rich α-2 glycoprotein 1 (LRG1) and G protein-coupled receptor 56 (GPR56) mRNA and protein levels. LRG1 promotes DN pathogenesis by enhancing TGF-β/activin receptor-like kinase 1 signaling in ECs (107). The overexpression of GPR56 reduced the phosphorylation and expression of eNOS, which may impair normal endothelial function (108). Additionally, elevated glucose levels lowered lysine methyltransferase 8 (SETD8) expression and increased myeloid zinc finger 1 (MZF1) expression. Their changes raise the expression of p-p65 and other endothelial inflammatory markers in hyperglycemic HGECs by adjusting Wingless-type family member 5 (WNT5A) transcription, which eventually causes damage to GECs (14). DN patients have higher levels of VCAM-1 and ICAM-1. Moreover, pro-inflammatory cytokines can bind to receptors on GECs, activating the NF-κB signal pathway (61, 109, 110). These results highlight the interactions between oxidative stress and inflammation in the context of hyperglycemia as major stimulators of DN endothelial dysfunction.

EndMT also plays a role in the initial phases of DN development as numerous fibroblasts in the kidneys of diabetics originate from the endothelium, which is triggered by TGF-β (111). Gremlin-1 actively upregulates the expression of TGF-β in DN, which results in a more severe development of kidney fibrosis (112, 113). Rho-associated kinase 1(ROCK1), an effector of TGF-β, is elevated in hyperglycemic environments and stimulates EndMT in DN (114). Ang-(1-7) treatment can normalize the levels of ROCK1 and ROCK2 in a diabetes-related context, providing a beneficial effect (115). In addition, ECs of the kidney are responsible for maintaining the glomerular filtration barrier (GFB). EndMT negatively affects the functional abilities of the glomerular filtration barrier (GFB) by decreasing the production of endothelial junctional proteins and increasing the deposition of extracellular matrix (ECM) proteins (116, 117). In early DN, ATP-binding cassette A1 (ABCA1) loss caused damage to glomerular endothelial cells through endoplasmic reticulum stress (ERS)-induced inflammation and apoptosis, which finally resulted in DN progression (118). Hyperglycemia significantly induced cytotoxicity and increased the expression of necroptosis markers in rat GECs. This result suggested that endothelial dysfunction induced by cell death can also partially explain the mechanisms underlying DN (119).

### 4.2 Diabetic retinopathy

Diabetic retinopathy (DR) is a common microvascular complication of diabetes mellitus and is the main cause of visual loss in the elderly (120). Two stages comprise the pathological process of diabetic retinopathy: non-proliferative (NPDR) and proliferative (PDR). Microvasculopathy in the DR retina is characterized by reduced capillary flexibility, heightened vascular permeability, localized inflammation, and the presence of growth factors that facilitate the development of neovascularization (95).

Hyperglycemia leads to the impairment of retinal endothelial cells (RECs) by ischemia, oxidative stress, and the release of pro-inflammatory factors. Patients with DR may experience increased vessel wall permeability and capillary occlusion as a result of increased adhesion molecule expression and decreased vasodilation found in retinal microvessels (121). Retinal binding protein 3 (RBP3) reduces inflammatory cytokines and inhibits VEGF’s activities in the retina, which may prevent the advancement of DR (122). In addition, excessive ROS (H_2_O_2_) accumulation in pathological situations such as hyperglycemia can activate the transcription factor HIF-1α, which increases the expression of inflammatory mediators and VEGF, damaging retinal microvessels (123, 124). Li et al. recently reported the anti-oxidative stress effect of F-box and WD repeat domain containing 7 (FBXW7) in ECs. FBXW7 alleviates harmful processes that lead to DR, such as DNA damage, mitochondrial dysfunction, ROS elimination, and PARP overactivation (125).

The death of RECs may accelerate the development of DR. In a hyperglycemic setting, increased homocysteine levels elevate the activity of dynamin-related protein 1 (Drp1) by causing its nitrosylation in RECs. This further fractures the mitochondria and elevates the apoptosis of RECs, finally aggravating diabetic retinopathy (126). Hyperglycemia-induced pyroptosis in human retinal microvascular endothelial cells (HRMECs) is characterized by increased caspase-1 activity, IL-β, NLRP1, NOX4, TXNIP, and NLRP3 expression (71). AGEs cause HRMECs and corneal ECs to undergo pyroptosis through cleaved caspase 1 and active gasdermin (GSDM), leading to blindness (127, 128). Hyperglycemia may promote TRIM46-mediated GPX4 ubiquitination, thereby lowering the expression of GPX4 in retinal capillary ECs, leading to more EC ferroptosis (129). RECs also help maintain the blood-retinal barrier (BRB). Loss of endothelial properties through EndMT affects REC barrier function and increased matrix protein deposition in mesenchymal cells may thicken the basement membrane, increasing vascular permeability in DR (39, 130, 131).

### 4.3 Diabetic neuropathy

Endothelial dysfunction often develops in the early stages of diabetes, impacting both the peripheral nervous system (PNS) and central nervous system (CNS), which can result in sciatic nerve irritation and diabetic neuropathy (132). DPN is a length-dependent injury of peripheral nerves that starts proximally in the feet and eventually moves to the hands. It is also a common consequence of diabetic microvascular disease (133). The thickening of the basement membrane and endothelial dysfunction with anomalies in endoneurium capillaries are characteristics of the diabetic nerve (133). Recent research has identified additional pro-oxidant enzymes, including NOXs, which are now known to be powerful ROS producers in DPN (134). Particularly, Nox2 and Nox4 are overexpressed in diabetic nerves and are involved in the degeneration of both structural and functional nerves (135, 136). Diabetic sciatic nerves contain less desert hedgehog. Inhibiting hedgehog signaling in ECs is sufficient to cause blood nerve barrier (BNB) breakdown and neuropathy. In addition, VEGFA increased BNB permeability induced by desert hedgehog deficiency and the severity of microangiopathy (137). Therefore, diabetic endothelial dysfunction in the neurovascular system is closely related to the state of oxidative stress.

### 4.4 Diabetic cardiomyopathy

Diabetic cardiomyopathy (DCM) is characterized by abnormal cardiac structure and function in the absence of cardiac risk factors in patients with diabetes, which can result in heart failure (HF) (138). According to a meta-analysis, diabetes reduces long-term survival and hospitalization in acute and chronic HF patients (139).

The development of diabetic cardiomyopathy is probably due to a combination of factors, such as endothelial dysfunction, glucose toxicity, mitochondrial dysfunction, and lipotoxicity (140). The diabetic heart has increased fatty acid oxidation but decreased glucose oxidation. It also exhibits elevated levels of pro-inflammatory cytokines and higher leukocyte infiltration (141, 142). Fibrosis is one characteristic of diabetic cardiomyopathy. EndMT can contribute to activated cardiac myofibroblasts. Therefore, in hyperglycemia, increased EndMT leads to excessive fibroblast activation and causes extracellular matrix protein overproduction, interstitial fibrosis, and a thicker basement membrane (143, 144). Moreover, both RAGE and AGEs increase in diabetes. Knocking out RAGE can decrease the degree of EndMT, accompanied by decreased expression of autophagy-related proteins (LC3BII/I and Beclin 1), and alleviate cardiac fibrosis in mice (145). The expression of mitochondrial calcium uptake 1 (MICU1) was decreased in the myocardial microvascular endothelial cells (CMECs) of diabetic mice. The reduction of MICU1 in diabetic CMECs showed a significant increase in inducible nitric oxide synthase (iNOS) enzymes related to nitrification stress and inflammation-related molecules. These changes led to exacerbated cardiac hypertrophy and fibrosis (31).

**Figure 5:**
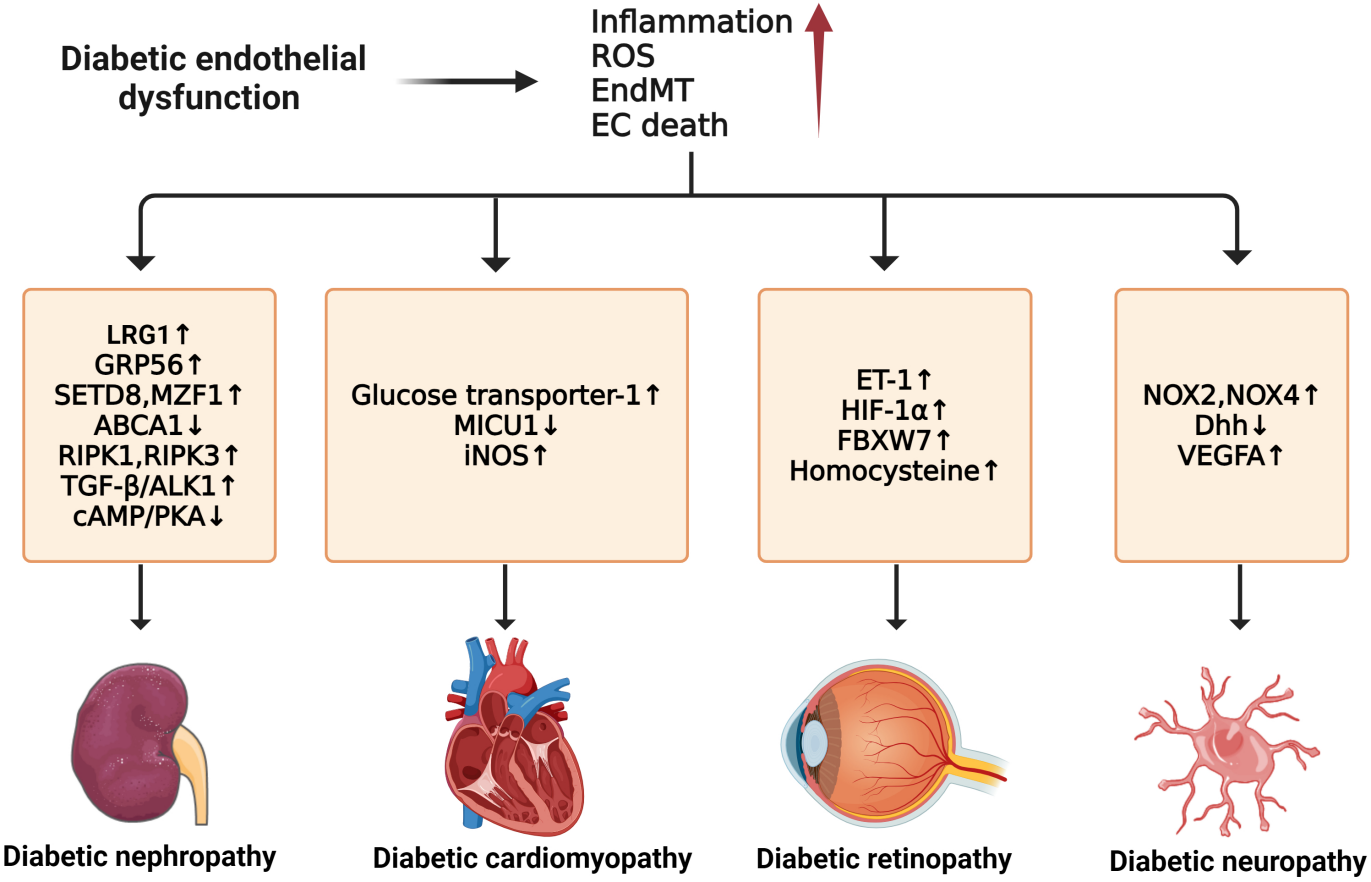
Molecular mechanisms of diabetic microvascular complications. In diabetic endothelial dysfunction, individuals are susceptible to microvascular complications such as diabetic retinopathy, diabetic nephropathy, diabetic neuropathy, and diabetic cardiomyopathy. The development of these complications is due to some common factors such as increased inflammation, ROS, EndMT, and endothelial cell death. For diabetic neuropathy, the upregulation of NOX2, NOX4, and VEGFA and the downregulation of Dhh can enhance its progression. Diabetic nephropathy can be accelerated by the upregulation of LRG1, GRP56, RIPK1, RIPK3, and TGF-β/ALK1 and the downregulation of ABCA1 and cAMP/PKA. In diabetic retinopathy, the exacerbations of ET-1, HIF-1α, FBXW7, and Hcy can worsen the condition. For diabetic cardiomyopathy, higher expression of GLU-1, MICU1, and iNOS contributes to its development.

Impaired NO signaling is closely correlated with diabetic cardiomyopathy. In diabetic rat hearts, eNOS level decreased in ECs when compared to non-diabetic controls (146). However, myocardial BH_4_ oxidation and NOS dysfunction are not always present in the cardiomyopathic phenotype or as early indicators of diabetes mellitus, suggesting a more significant role of vascular endothelium in diabetic cardiomyopathy (147). Moreover, substantial inflammatory reactions in diabetic cardiac tissue cause elevated levels of IL-1β, IL-18, IL-6, and TNF-α. EPC dysfunction leads to adverse remodeling in the diabetic heart, which can be improved by bone morphogenetic protein-7 (BMP-7) (148). Though the role of ECs in diabetic cardiomyopathy is not directly confirmed, there has been extensive discussion on the crosstalk between ECs and cardiomyocytes, especially their metabolisms and angiogenesis functions mediated by VEGF (149). For instance, a study has suggested that cardiomyocyte-derived exosomes can modulate endothelial glucose transport and metabolism by sending glucose transporters and the associated glycolytic enzymes to the ECs (150). Thus, adverse changes in cardiomyocytes can indirectly affect myocardial ECs by their metabolism interaction. Nevertheless, a deeper understanding of ECs in diabetic cardiomyopathy still needs further investigation (**Figure 5**).

## 5 Current therapies

### 5.1 Metformin

Metformin is a first-line therapy for treating type 2 diabetes due to its robust and pleiotropic effects on glucose metabolism. Metformin can protect the endothelium beyond lowering blood glucose levels. It has various effects on ECs (3, 4). Improved endothelium-dependent vasodilation from *in vivo* and *in vitro* studies showed a positive impact of metformin treatment on vascular endothelial function in diabetes (151).

Metformin can regulate the expression of inflammatory markers and slow the progression of diabetic vascular complications, including DN and PAD, by inhibiting inflammation in diabetes. It also has a favorable impact on certain cardiovascular risk markers (152, 153). Metformin inhibits NF-κB activation via AMPK activation and blockade of the PI3K-Akt pathway, which reduces the expression of proinflammatory and adhesion molecule genes (154, 155). *In vivo* investigations found that metformin can prevent diabetic nephropathy by reducing LRG1 and TGF-β1/ALK1-induced kidney angiogenesis and decreasing inflammatory cytokine levels (156). Moreover, metformin prevented the induction of femoral artery ultrastructural changes and the expression of vascular AGEs, ET-1, and iNOS, dyslipidemia. This resulted in an improvement in lower extremity arterial endothelial function by reducing the inflammation state in a rat model of diabetes-induced lower extremity arterial disease (LEAD) for 12 weeks. This resulted in an improvement in lower extremity arterial endothelial function (157). Metformin decreases the production of ROS from mitochondria by stimulating both AMPK-dependent and AMPK-independent pathways in human leukocytes and ECs (158, 159). It also regulates EC function, survival, proliferation, and senescence through the SIRT1 and LKB1/AMPK pathways (95). It restores Hedgehog pathway activity to suppress hyperglycemia-induced autophagosome production in diabetic retinal vasculature and cultured HUVECs (160). Metformin has been shown to protect vascular ECs by enhancing autophagy flux and diminishing lipid accumulation (161).It attenuated the development of diabetes-accelerated AS by reducing Dynamin-related protein (Drp1)-mediated mitochondrial fission in an AMPK-dependent manner (162).

However, metformin has some noticeable side effects, such as nausea, vomiting, bloating, a metallic taste in the mouth, abdominal pain, cramps, and changes in intestinal motility, which may result in loose stools and diarrhea that can be challenging to manage. Around 25% of patients experience gastrointestinal side effects and about 5% are unable to tolerate metformin (163). Additionally, metformin has been linked to increased levels of homocysteine and vitamin B12 deficiency, which can exacerbate diabetic neuropathy (164).

### 5.2 Dipeptidyl peptidase-4 inhibitors

Dipeptidyl peptidase-4 inhibitors (DPP-4i) are commonly used treatments for patients with T2DM and include vildagliptin, linagliptin, sitagliptin, teneligliptin, and others. It blocks the breakdown of glucagon-like peptide-1 (GLP-1) and glucose-dependent insulinotropic polypeptide (GIP) to decrease blood glucose levels. In the vascular system, DPP4 is widely expressed by ECs, cardiomyocytes, and many other cell types (165), implying its role in diabetic vascular disease.

Increasing data suggests DPP-4i inhibitors have a protective role in endothelium and AS, independent of their hypoglycemic effects (3, 165). DPP-4i treatment decreased carotid intima-media thickness (IMT) in individuals with diabetes, which slowed the development of AS (166). Vildagliptin prevents hyperglycemia-induced endothelium damage in a GLP-1-independent way. It directly activates TRPV4 to promote Ca^2+^ absorption, AMPK activation, and SIRT1. In this way, it promotes endothelial-dependent vasorelaxation to protect against hyperglycemia-induced endothelial dysfunction (167). Sitagliptin’s activation of AMPK prevented the formation of ROS, the collapse of mitochondrial membrane potential, and the apoptosis of HUVECs induced by high glucose (168). It triggered the AMPK/unc-51-like autophagy activating kinase 1 signaling pathway to restore basal autophagy in EPCs, preventing EPC apoptosis. Thus, it preserves EPC angiogenic function, improving diabetic ischemia angiogenesis (169). In hyperglycemia, teneligliptin enhances HUVEC proliferation and inhibits HUVEC apoptosis by promoting B-cell lymphoma 2 (BCL2) expression, decreasing proapoptotic genes (*BAX* and *CASP3*), and expressing cell-cycle inhibitor hallmarks (P27, P21, and P53) (170). Adenosine AMPK phosphorylation by DPP4 promotes vascular ECs apoptosis and autophagy, while microRNA 5680 inhibits this process as a DPP4i (171).

DPP-4i have a significant impact on the development and progression of diabetic vascular disorders. It protects brain microvascular ECs against high-glucose and hypoxic conditions (172). Linagliptin effectively inhibits TNF-α-induced NF-κB nuclear protein p65 accumulation and promotes activation in RECs. It also reduces TNF-α-induced vascular cytokine production, such as IL-6, IL-8, ICAM-1, and VCAM-1 (173). In diabetic kidney ECs, linagliptin inhibited TGF-β2-induced EndMT by suppressing levels of microRNA 29s (174). It can cause a decrease in endothelial toll-like receptors 2 (TLR2) expression and a subsequent increase in NO bioavailability. Linagliptin reduced ET-1-induced basilar arteries contraction in diabetic rats, improving diabetic cerebrovascular dysfunction (175). In addition, evogliptin directly interferes with pathological retinal neovascularization (NV) by blocking VEGF-induced adenosine 5′-diphosphate ribosylation factor 6 (Arf6) activation in ECs (176).

Nevertheless, research has shown an increased risk of heart failure with DPP-4i, especially saxagliptin. Patients with moderate renal dysfunction were found to have an increased risk of heart failure. Further research is necessary to comprehend the impact of declining renal function on the cardiovascular safety of DPP-4 inhibitors (177). Furthermore, the use of sitagliptin increases the odds ratio for reported pancreatitis by a factor of six. Additionally, there was an increase in reported cases of pancreatic and thyroid cancer compared to other therapies. These findings suggest that further studies on the effects of DPP-4i on the thyroid gland and exocrine pancreas in humans are necessary (178).

### 5.3 Glucagon-like peptide 1 receptor agonists

Glucagon-like peptide 1 (GLP1) is a type of incretin hormone released from gut endocrine cells (179). It regulates blood glucose levels by enhancing insulin secretion in pancreatic β cells and reducing glucagon release (180). Glucagon-like peptide 1 receptor agonists (GLP-1RA) are artificial mimics or analogs of human GLP-1, including exenatide and liraglutide. The cardiovascular benefits of GLP-1RA in patients with T2DM and established cardiovascular diseases have been indicated through many clinical trials (179, 181).

Previous studies have demonstrated that GLP-1RA have the ability to enhance endothelial function in diabetic patients (3, 179, 182). Exenatide decreased high-glucose-induced ROS production and the apoptotic index in CMEC. This protective effect is reliant on the downstream suppression of Rho via a cAMP/PKA-mediated pathway (183). Additionally, it protects ECs from oxidant stress through reducing autophagy, which is dependent on the restoration of histone deacetylase 6 (HDAC6) in a GLP-1R-extracellular signal-regulated kinase (ERK)1/2-dependent way (184). *In vivo* and *in vitro* studies show that exendin-4 decreases HHcy-induced ER stress and enhances endothelial function by upregulating endoplasmic reticulum oxidoreductase (ERO1α) via AMPK in ECs and arteries. AMPK activation improves EC protein folding to reduce ER stress (185). In addition, liraglutide prevents hyperglycemia-induced Smad2 phosphorylation and EndMT via the AMPK pathway, indicating the prevention of liraglutide towards hyperglycemia-induced EndMT (186). The GLP-1 metabolite GLP-1(9-36) amide enhanced human aortic EC viability in response to hypoxic injury and hydrogen peroxide treatment via a NO- and mitochondria-dependent mechanism (187). Large-scale cardiovascular outcome trials have shown that GLP-1RAs improve cardiovascular outcomes in T2DM patients with high cardiovascular risk or established atherosclerotic cardiovascular disease (181, 188, 189), mainly through their anti-atherosclerotic properties. T2DM increases carotid intima-media thickness (CIMT), while CIMT decreases in the GLP-1RA-treated group, suggesting a slowing of the atherosclerotic process (190). In the context of ischemic cardiac injury, GLP-1RA’s cardioprotective actions mainly target GLP-1R in mouse Tie2+ endothelial cells. The cardioprotective functions of liraglutide were attenuated in Glp1r^Tie2-/-^ mice (191). The cellular localization of GLP-1R in the heart may provide insight into the mechanism of GLP-1RA in diabetic MI and other cardiovascular complications. In a concentration-dependent way, liraglutide improved the function of hyperglycemia-treated HUVECs and phosphorylated Akt, eNOS, and ERK1/2 *in vitro* and *in vivo*. Therefore, liraglutide may help treat hind-limb ischemia in type 2 diabetic mice (192).

Current findings indicate that GLP-1RA have a favorable safety profile and do not increase cardiovascular risk in patients with T2DM. Ongoing trials will further assess their cardiovascular effects (193). However, exenatide and sitagliptin have similar side effects that can increase the risk of pancreatitis, thyroid cancer, and pancreatic cancer. When taken together, there is a significantly increased association with these therapies and the development of thyroid and pancreatic cancer (178). GLP-1 RA were found to be significantly associated with gastrointestinal adverse events. Among these, semaglutide had the highest risk of nausea, diarrhea, vomiting, constipation, and pancreatitis, while liraglutide had the highest risk of upper abdominal pain (194).

### 5.4 Sodium-glucose cotransporter 2 inhibitor

Approximately 97% of the total renal glucose reabsorption is attributed to SGLT2 (195). SGLT2i inhibits this high-capacity glucose transporter, SGLT2, in the proximal convoluted tubule and helps the kidneys excrete glucose in the urine to lower blood glucose (196). SGLT2i improved obesity, aberrant lipid metabolism, inflammation, endothelial dysfunction, and nephropathy in diabetic mice after 4-week repeated dosing (197). The main types of SGLT2i are canagliflozin, dapagliflozin, and empagliflozin.

Based on numerous clinical trials, patients with diabetic chronic heart failure and CAD showed marked improvement in their flow-mediated dilation (FMD) and blood pressure after empagliflozin and canagliflozin treatments (198). Moreover, several types of SGLT2i prevented diabetic nephropathy in rodents (199). El-Daly et al. suggest that hyperglycemia-induced oxidative stress affects protease-activated receptor 2-mediated vasodilation in the endothelium through a NOX-triggered signaling cascade, which can be suppressed by SGLT2i (200). Furthermore, the inhibition of mitochondrial fission induced by empagliflozin protected cardiac microvascular endothelial cell barrier function by suppressing mtROS production and oxidative stress, thereby preventing senescence of cardiac microvascular endothelial cells. Furthermore, it helps to maintain eNOS phosphorylation, endothelium-dependent relaxation, and the integrity of heart microvascular barrier (201). *In vivo* studies showed the improvement of endothelial dysfunction in diabetic ApoE^-/-^ mice after treatment with empagliflozin. This is due to the anti-inflammatory effect of empagliflozin, which decreases the levels of TNF-α, ICAM-1, and vasoconstrictive eicosanoids (202). In addition, empagliflozin significantly increased the levels of the Beclin1 protein, the LC3B-II/I ratio, and the p-AMPK protein, inducing autophagy in HUVECs via the AMPK signaling pathway, which delays the evolution of AS (203). Li et al. found that empagliflozin reduced EndMT in the proximal tubule and ECs in diabetic mice, decreasing kidney fibrosis and potentially protecting the kidneys (204). Oral empagliflozin improved coronary endothelial function by increasing L-arginine/asymmetric dimethylarginine ratio and NO bioavailability (205).

In addition, other types of SGLT2i also improve endothelial function through different mechanisms. The Canagliflozin and Renal Events in Diabetes with Established Nephropathy clinical evaluation trial showed that SGLT2 blockade slows the progression of established diabetic kidney disease and reduces the risk of cardiovascular death, myocardial infarction, stroke, and hospitalization due to heart failure in T2DM patients (206). In human coronary artery ECs from donors with DM (D-HCAECs) exposed to TNF-α, canagliflozin reduces inflammasome activation and ROS generation. It reduces inflammasome activation by inhibiting intracellular Ca^2+^ and extracellular signal regulated kinase (ERK) 1/2 phosphorylation (207). Additionally, dapagliflozin reduces endoplasmic reticulum (ER) stress in human coronary artery endothelial cells (208).

However, SGLT-2i have common side effects, such as urinary tract infections, euglycemic ketoacidosis, orthostatic hypotension, dehydration, and cardiovascular adverse events (209). Additionally, they are associated with increased risks of diabetic ketoacidosis and lower-limb amputations. The risk of lower-limb amputations is higher in patients with a history of amputation and peripheral vascular disease. Dapagliflozin is specifically associated with a high risk of toe amputation (210, 211). In general, SGLT2i are a beneficial treatment option for type 2 diabetes. However, their use should be thoroughly assessed and supervised to minimize potential adverse effects.

Taken together, conventional therapeutic approaches have distinct targets aimed at alleviating endothelial dysfunction in the context of diabetic vascular complications. Here we focus on the potential benefits of metformin, DPP-4 inhibitors, GLP-1 receptor agonists, and SGLT-2 inhibitors in the improvement of diabetic vascular disorders, especially their ability to improve inflammation, oxidative stress, cell death, and EndMT. Additionally, we discuss some common side effects of these therapies that should be noted. The objective is to identify additional therapeutic applications for these medications in treating various types of diabetic vascular complications.

## 6 Future directions and novel regulators

### 6.1 MicroRNA therapies

MicroRNAs (MiRNAs) are short, endogenous, non-coding RNAs with a normal length of 22 nucleotides that regulate gene expression post-transcriptionally. They target more than half of the transcripts that code for proteins, making them implicated in almost all animal developmental and pathological processes (212). Research indicates that they are important for preserving optimal vascular homeostasis and preventing the sequelae of diabetes-induced end-organ destruction. MiRNAs have been shown to regulate endothelial dysfunction in many aspects and also regulate the diabetic microvasculature in many diabetes-associated complications (213).

In human aortic endothelial cells, high glucose and thrombin reduce miR-146a expression when combined. Overexpression of the miR-146a mimic can downregulate IL-6 and IL-8 in hyperglycemia/thrombin-stimulated human aortic endothelial cells (HAECs) (214). Moreover, miR-146a CAN rescue senescent HUVECs. *In vivo* and *in vitro* results demonstrate that miR-146a-5p mimics inhibit endothelial interleukin-1 receptor-associated kinase-1 (IRAK-1) and ICAM-1 expression, indicating an anti-inflammatory function in vessels (215). MiR-30 promotes oxidative stress, lipid peroxidation, and endothelial dysfunction in cultured ECs by regulating exogenous fatty acid oxidation. Thus, it might represent a potential therapeutic target for diabetic microvascular dysfunction (216). Mir-20a-5p reverses the expression of high phosphatase, tensin homolog, and autophagy-related 7 and promotes AKT and mTOR phosphorylation in high glucose circumstances, inhibiting EPC autophagy and apoptosis (217). This suggests that mir-20a-5p has a protective effect against cell death induced by hyperglycemia.

The evolving role of miRNAs in diabetic vascular complications has become a hot topic in recent years. In the glomerular vascular ECs of the kidney of DN patients, the expression of miR-155 was increased. Human renal glomerular Ecs undergo an inflammatory response and apoptosis due to negative regulation of miR-155 by E26 transformation-specific sequence 1 (ETS-1) and its downstream components VCAM-1, MCP-1, and cleaved caspase-3 (218, 219). Overexpression of miR-375 in ECs increases proliferation, wound closure, spheroid sprouting, and tube formation by targeting Kruppel-like factor 5 via altering phospho-p65 NF-κB signaling in diabetic critical limb ischemia (220). Overexpression of miR-181a-5p in HUVECs impaired the endothelial barrier and decreased gene expression of OCLN variants 1 and 2, the genes that meditate occludin expression. Occludin downregulation in various organs may cause DR or DN due to elevated miR-181a-5p levels (221). In DR, research has shown that miR-15b-5p inhibits the expression of COL12A1, a gene encoding collagen type XII α 1 chain, and inhibits the proliferation, migration, and angiogenesis of hyperglycemia-induced human retinal vascular endothelial cells (222). HG induced pyroptosis in a cell culture model of DR, whereas miR-590-3p inhibited pyroptosis by targeting the NLR family pyrin domain containing 1 (NLRP1) and inactivating the NOX4 pathway (71). In human retinal microvascular endothelial cells cultured in high glucose, miR-93 expression increases dramatically, while the overexpression of miR-93 exacerbates ferroptosis by increasing ROS generation and causing Fe^2+^ accumulation (223). MiR-126 overexpression can reduce EC apoptosis and promote angiogenesis by modulating VEGFR2 signaling. Consequently, it may ameliorate ischemic stroke in diabetic mice (224).

However, the use of miRNA technology at the clinical level is a potential challenge due to certain physiological barriers that affect their stability. The advanced drug delivery systems such as nanoparticles, however, have emerged as novel carriers for miRNA targeting to help solve this. There are still many problems such as the complexity of advanced drug delivery systems, especially for lipid-based nanocarriers, and the concerns of safety of the substance incorporated in these systems (225). Nanoparticles also cause possible side effects including inflammatory reactions, oxidative stress, and cell apoptosis, leading to cytotoxicity (226). MiRNAs also have the potential to target multiple genes, which can lead to unintended consequences and off-target effects. Another challenge is the complexity in the tissue and cell-specific expression and functions of specific miRNAs in the same diabetic complication. This could be related to the cell-type specific patterns, different model systems and animals studied, time of sampling, or the severity of the complications in the models studied (227).

### 6.2 Stem cell therapies

Stem cell-based therapy is an important field of regenerative medicine that regulates endogenous stem cells to enhance tissue homeostasis and regeneration (228). Mesenchymal stem cells (MSCs) are the main cell source used in stem cell-based therapy. MSC-based cell therapy has been recognized as an effective treatment for diabetes mellitus and its complications, serving as a means of preventing diabetic damage to the vascular endothelium (229, 230). As MSCs have long been considered a key source of cells in regenerative medicine, we will also discuss the effects of MSC-derived exosomes and extracellular vesicle (EVs) on diabetic endothelial dysfunction.

*In vitro*, human umbilical cord-derived MSCs restored high glucose-damaged HUVEC survival, wound healing, migration, angiogenesis, and senescence through a MAPK/ERK signaling mediated paracrine effect (230). Delivery of circ-Snhg11 from hypoxia-pretreated adipose-derived stem cell-originating exosomes (ADSC-HExo)-embedded GelMA hydrogels (GelMA-HExo) can improve EC survival and function by activating miR-144-3p/NFE2L2/HIF1α signaling, implicating the role of ADSC-Exos in diabetic wound healing (231). Similarly, exosomes from atorvastatin-pretreated MSCs may improve endothelial cell function via the AKT/eNOS pathway by upregulating miR-221-3p (232). MSC-derived small extracellular vesicles (sEV) therapy enhanced angiogenesis, migration, and proliferation in senescent HUVECs. It also ameliorated mitochondrial dysfunction and reduced ROS levels while providing protection against senescence-related problems. Its subcutaneous injection can promote skin wound healing in an animal model of aging and type-2 diabetes (233).

The increasing number of studies focusing on miRNA and stem cells holds the potential to improve the treatment of diabetic complications by providing a more fundamental approach (**Table 1**). However, it is important to note that translating stem cells research into therapies is complex and challenging. Uncontrolled proliferation of transplanted stem cells may lead to tumor formation in the treatment of diabetes. Improving stem cell efficacy is also a challenge, though there has been discussion about enhancing their differentiation potential through coculture techniques to address this (234). Stem cell translation may also lead to an unavoidable immune response. Research is ongoing on gene editing and nuclear transfer techniques in stem cells to help stem cells escape the immune response in the human body (235). Overcoming these obstacles through further research is critical to the development of effective novel therapies in the future.

**Table 1:** Regulators with endothelium-protective effect in diabetic complications.

Diabetic complication	Therapy	Mechanism	Reference	
CAD	GLP-1RA	Anti- atherosclerosis	(191)
	SGLT2i	Reduce ER stress Suppress mtROS	(209) (202)
Cerebrovascular disease and stroke	DPP-4i	Decrease TLR2	(176)
		MiR-126	(225)
	Metformin	Suppress inflammation, AGEs, and ET-1	(158)
	GLP-1RA	Phosphorylate Akt and eNOS	(193)
Peripheral artery disease	ADSC-Exos	Activate miR-144-3p/NFE2L2/HIF1α signal	(232)
	Metformin	Suppress inflammation, oxidative stress, and fibrosis	(154, 157)
	DPP-4i	Inhibit EndMT	(175)
Diabetic nephropathy	SGLT2i	Inhibit EndMT	(205)
	Metformin	Suppress autophagosome	(161)
	DPP-4i	Suppress inflammation and Arf6 activation	(174, 177)
Diabetic retinopathy	MiR-15b-5p	Inhibit COL12A1	(223)
	MiR-590-3p	Inhibit pyroptosis	(72)

## 7 Conclusions

Significant advancements have been made in the study of endothelial dysfunction in diabetes and its associated vascular complications. This review has discussed the fundamental mechanisms of diabetes and its associated vascular disorders, including inflammation, oxidative stress, cell death, and EndMT. Based on the findings of these studies, it is evident that there are many promising targets and therapies, such as metformin, DPP-4i, GLP-1RA, SGLT2i, miRNA, and stem cells, which hold potential for clinical use. In contrast to the more well-known processes of inflammation and oxidative stress, the mechanisms of diabetic endothelial dysfunction underlying cell death and EndMT remain poorly understood. Furthermore, there are still numerous limitations and side effects of the traditional therapies and novel regulators targeting diabetic vascular complications (**Table 2**). Therefore, given the uncertainties surrounding these variables and the intricate nature of alterations in diabetic endothelial dysfunction, further research is warranted to elucidate these mysteries.

**Table 2:** The targets and side effects or limitations of diabetic vascular complications regulators.

Therapy	Targets	Reference	Side effects or Limitations	Reference
Metformin	PAD	(153, 158)	Gastrointestinal diseases	(164)
	DN	(154, 157)	Vitamin B12 deficiency	(165)
	DR	(161)	Increased Hcy	(165)
DPP-4i	DR	(174, 177)	Heart failure	(29)
	DN	(175)	Pancreatitis	(179)
	Cerebrovascular disease	(176)	Pancreatic cancer	(179)
			Thyroid cancer	(179)
GLP-1RA	PAD	(193)	Pancreatitis	(179)
	Cardiovascular disease	(180, 192)	Thyroid cancer	(179)
			Pancreatic cancer	(179)
			Gastrointestinal diseases	(195)
SGLT2i	CAD	(199, 208)	Ketoacidosis	(211, 212)
	DN	(200, 205, 207)	Lower-limb amputations	(210)
	AS	(204)	Urinary tract infections	(210)
	Cerebrovascular disease	(207)	Cardiovascular risk	(210)
MiRNA	PAD	(221)	Instable	(226)
	DN	(219, 220)	Unsafe drug delivery system	(226, 227)
	DR	(72, 223)	Off-target effects	(228)
	Cerebrovascular disease	(225)	Pleiotropy	(228)
Stem cell	PAD	(231, 232, 234)	Tumor formation	(235)
			Inefficient	(235)
			Immune response	(236)

## Author contributions

D-RY: Visualization, Writing – original draft, Writing – review & editing. M-YW: Writing – review & editing, Writing – original draft. C-LZ: Funding acquisition, Writing – review & editing. YW: Conceptualization, Funding acquisition, Supervision, Visualization, Writing – review & editing.
